# Treatment perspectives and concerns among pediatric cleft lip and palate patients: a cross-sectional study

**DOI:** 10.1186/s12903-025-06693-w

**Published:** 2025-08-12

**Authors:** Jeannie Rachel Manohar, Joe Mathew Cherian, Mebin George Mathew, Abi M. Thomas

**Affiliations:** Department of Pedodontics and Preventive Dentistry, Christian Dental College, Ludhiana, Punjab 141008 India

**Keywords:** Cleft lip and palate, Pediatric dentistry, Facial aesthetics, Speech challenges, Emotional well-being, Interdisciplinary care, Appliance therapy

## Abstract

**Background:**

Children with cleft lip and palate (CLP) often face physical and emotional challenges impacting their quality of life. This study aims to assess the treatment outlook and concerns of school-going CLP patients from the perspective of pediatric dentistry in a tertiary healthcare setting in North India.

**Methods:**

A cross-sectional, questionnaire-based study was conducted on 30 patients undergoing appliance therapy for CLP. The questionnaire, available in English and regional languages, included sections on general information, treatment, aesthetic, functional, and emotional aspects. Responses from children and their parents were analyzed, with chi-square tests used to evaluate associations between variables.

**Results:**

Among participants, 76.6% were referred to pediatric dentists by surgeons, and 66.7% of those aged over 12 years had been receiving dental treatment for more than three years (*P* = 0.031). Dissatisfaction with facial appearance was reported by 56.7% of participants, with significant speech dissatisfaction noted particularly among children under 12 years (66.7%, *P* = 0.020). Emotional and social challenges included 56.7% struggling with oral recitation at school, affecting 75% of adolescents (*P* = 0.098). Furthermore, 46.7% expressed reduced confidence with intraoral appliances, especially males (53.3%).

**Conclusions:**

This study highlights the crucial role of pediatric dentists in the comprehensive care of children with CLP, emphasizing the need for personalized dental treatment and interdisciplinary collaboration. Addressing functional and emotional well-being is essential to improve overall outcomes and quality of life for these patients.

**Trial registration:**

Not applicable.

**Supplementary Information:**

The online version contains supplementary material available at 10.1186/s12903-025-06693-w.

## Introduction

Cleft lip and/or palate (CLP) is one of the most common congenital anomalies affecting the craniofacial region, characterized by an opening or split in the upper lip and/or the roof of the mouth. Management of CLP is complex and requires long-term, multidisciplinary care that often extends from infancy through adolescence and into young adulthood [1, 2]. The primary goals of CLP treatment are to facilitate normal craniofacial growth, achieve functional orofacial closure, and improve the patient’s ability to communicate effectively, focusing on speech, hearing, and facial expression, which collectively enhance social interaction and quality of life [2].

A comprehensive care team includes surgeons, orthodontists, speech therapists, and pediatric dentists, all dedicated to delivering both functional and aesthetic outcomes to foster an improved quality of life for patients [3]. Pediatric dentists play a crucial role in the CLP care team by providing essential dental and oral health support, taking into account various patient and familial factors such as cultural background, socioeconomic status, prior healthcare experiences, and individual expectations. Recognizing these diverse needs is essential for delivering care that goes beyond the physical aspects of CLP to address the psychological and social dimensions of health [4].

Oral Health-Related Quality of Life has emerged as a key measure in CLP management, reflecting the importance of patient satisfaction with treatment and its effects on daily life [3]. Adolescence and young adulthood often mark critical stages for evaluating treatment success, as growth and adherence to long-term therapy typically yield more definitive outcomes [5]. During this period, patient maturity and commitment to care regimens allow clinicians to assess not only clinical improvements but also the psychosocial impacts of CLP therapy [4]. This holistic evaluation extends beyond functional and aesthetic aspects, considering the patient’s self-image, speech, social integration, and overall quality of life [3, 4].

Effective CLP care hinges on understanding and responding to the wide spectrum of patient and parental concerns, fostering collaboration and shared decision-making with the pediatric dentist and the broader healthcare team [6]. Addressing these concerns is essential for delivering comprehensive care that meets the physical, psychological, and social needs of patients with CLP [7].

A comprehensive understanding of the treatment outlook and concerns of patients with CLP, along with their families, is essential for achieving optimal outcomes and fostering effective collaboration with pediatric dentists. This study was conducted to evaluate these diverse treatment-related perspectives, aiming to address gaps in care and improve the overall quality of CLP management.

## Methodology

### Study design and setting

This cross-sectional study was started after obtaining ethical clearance from the institutional ethics committee (BMHR-IEC/Apprvl-R-Proj/23-04-123/CDC).

### Participants

A total of 30 patients undergoing appliance therapy for CL/P were recruited from the department. Both the patients and their parents participated by completing questionnaires designed to evaluate dental health and quality of life, specifically measuring the impact of oral health on overall well-being.

### Inclusion criteria

- Patients aged between 5 and 16 years.
- Diagnosed with unilateral or bilateral CLP.
- Undergoing appliance therapy in the department for at least one month.
- Willingness to participate in the research study.

### Exclusion criteria

- Patients with systemic conditions or special healthcare needs.
- Failure to obtain written informed consent from parents or guardians.

### Ethical considerations

Written informed consent was obtained from the parents/guardians after a thorough explanation of the study’s objectives and procedures. Additionally, written assent was obtained from participants aged 12 years and older, adhering to ethical guidelines for research involving minors.

### Data collection

Previous dental records of the patients were reviewed to supplement questionnaire data. To ensure comprehension and accurate responses, questionnaires were provided in English and regional languages based on the participants’ preferences. Both patients and their parents completed the questionnaires independently to capture distinct perspectives.

### Questionnaire development

The questionnaire used in this study was adapted from a previously published instrument by Nor JH [8] which explored the attitudes and concerns of patients with cleft lip and palate and their caregivers. Modifications were made to suit the pediatric dental context and enhance cultural relevance within the Indian population. The questionnaire was meticulously designed to assess various dimensions affecting the quality of life in patients with CLP and consisted of five sections:

1. General Information and Socioeconomic Status
  ◦ Collected demographic data such as age, gender, and place of residence.
  ◦ Assessed socioeconomic status using a standardized scale [3].

2. Treatment Aspects
  ◦ Gathered information on the duration of treatment and type of appliance therapy (fixed or removable).
  ◦ Explored adherence to treatment and any difficulties experienced during therapy.

3. Aesthetic Aspects
  ◦ Evaluated perceptions of facial appearance before and during treatment.
  ◦ Identified any noticeable changes and main concerns regarding aesthetics.

4. Functional Aspects
  ◦ Assessed the impact of appliance therapy on speech, eating, and sleeping patterns.
  ◦ Investigated any functional impairments or improvements due to the treatment.

5. Emotional and Social Aspects
  ◦ Explored emotional well-being, including self-confidence and feelings related to the appliance.
  ◦ Examined social interactions, such as making friends, participation in school activities, sports, and experiences of teasing or bullying.

#### Questionnaire Validation

The questionnaire was rigorously validated to ensure reliability and appropriateness for assessing treatment concerns of patients with CLP. A panel of six experts in pediatric dentistry, psychology, and public health reviewed the questionnaire for clarity, relevance, and comprehensiveness. Based on their feedback, items were refined to better align with study objectives.

A pilot study was conducted with 10 participants to assess comprehension and ease of response. Feedback from this group led to minor adjustments, enhancing the questionnaire’s clarity. Internal consistency was measured using Cronbach’s alpha, yielding a value of 0.85, indicating strong reliability. To assess stability, test-retest reliability was conducted with a subset of participants after a two-week interval, resulting in an intraclass correlation coefficient of 0.90, confirming consistency over time.

This validation process established the questionnaire’s reliability and suitability for evaluating the treatment-related perspectives of patients with cleft lip and palate and their families.

### Data analysis

Responses from the questionnaires were coded and entered into a SPSS version 23.0. Descriptive statistics were used to summarize demographic information and questionnaire responses. Inferential statistics, such as chi-square tests for categorical variables and t-tests for continuous variables, were employed to identify significant associations between treatment aspects and quality of life measures. A p-value of less than 0.05 was considered statistically significant.

## Results

Over the six-month study period, 30 children diagnosed with CLP were enrolled, comprising an equal number of males and females (15 each). The participants ranged in age from 5 to 16 years, with 60% (*n* = 18) under 12 years of age and 40% (*n* = 12) aged 12 years or older. Detailed demographic information is presented in Table 1.

**Table 1:** Demographics of the participants

Sr. No.	Parameters		*n*	%
1.	Age	<=12	18	60.0
		13–16	12	40.0
2.	Gender	Male	15	50.0
		Female	15	50.0
3.	School	Government	5	16.7
		Private	25	83.3
4.	Socio-Economic Status	Upper (I)	1	3.3
		Upper Middle (II)	5	16.7
		Lower Middle (III)	13	43.3
		Upper Lower (IV)	10	33.3
		Lower (V)	1	3.3

A significant majority of participants (76.6%, *n* = 23) were first introduced to a pediatric dentist through referrals from surgeons involved in their CLP surgical treatment. Among the older age group (≥ 12 years), 66.7% (*n* = 8) had been undergoing dental treatment for more than three years, a statistically significant finding (*P* = 0.031). Overall, 90% (*n* = 27) of the children were receiving fixed appliance therapy. Specifically, 53.3% (*n* = 16) were undergoing arch expansion therapy using a quad helix appliance, while 26.7% (*n* = 8) were receiving skeletal expansion therapy with a face mask. These treatment details are summarized in Table 2.

**Table 2:** Treatment aspect

Q. NO.	Questions	Answers	N	%
			30	100
1	Who referred you to a pediatric dentist for cleft lip and palate treatment?	Surgeon	23	76.7
		Orthodontist	0	0
		Speech Therapist	0	0
		Others	7	23.3
2	How long has it been since you are visiting a pediatric dentist?	Less than 1 year	6	20.0
		More than 1 year	8	26.7
		More than 2years	3	10.0
		More than 3years	13	43.3
3	What treatment are you undergoing at present?	Arch expansion	16	53.3
		Skeletal Correction	8	26.7
		Teeth alignment	4	13.3
		Prosthetic rehabilitation	2	6.7
4	What type of appliance are you using now?	Fixed Appliance	27	90.0
		Removable Appliance	3	10.0
5	Which group has been most important in your care?	Surgeon	23	76.7
		Orthodontists	3	10.0
		Pediatric Dentist	23	76.7
		Speech therapists	1	3.3

More than half of the participants (56.7%, *n* = 17) expressed dissatisfaction with their current facial appearance. Female participants reported higher levels of concern (66.7%, *n* = 10) compared to males. Additionally, 70% (*n* = 21) of the children noticed noticeable changes in their appearance during treatment, with dental changes being the most significant. As treatment progressed, concerns about dental aesthetics remained the primary focus for many participants. Detailed findings on aesthetic concerns related to the nose, lips, jaws, and teeth are provided in Table 3.

**Table 3:** Aesthetic aspect

Q. NO.	Questions	Answers	*N*	%
1	How satisfied are you with your facial appearance at the moment?	Very satisfied	0	0
		Satisfied	8	26.7
		No feelings	5	16.7
		Dissatisfied	17	56.7
		Very dissatisfied	0	0
2	Is there a noticeable change in your appearance from the beginning of your treatment until now? If yes, then which part of your face has changed?	Nose	1	3.3
		Lips	5	16.7
		Jaws	14	46.7
		Teeth	21	70
3	Which part of your face is your main concern at present?	Nose	16	53.3
		Lips	8	26.7
		Jaws	14	46.7
		Teeth	21	70

Speech difficulties emerged as a prominent issue among the participants. Half of the children (50%, *n* = 15) were dissatisfied with their speech. This dissatisfaction was particularly prevalent among children under 12 years of age (66.7%, *n* = 12; *P* = 0.020) and among female participants (60%, *n* = 9). Despite these challenges, 53.5% (*n* = 16) believed that the treatment provided by the pediatric dentist had improved their speech.

Notably, 60% (*n* = 18) of the participants had not received formal speech therapy. Among those who did receive speech therapy, only 20% (*n* = 2) expressed satisfaction with the outcomes. Dietary restrictions due to appliance therapy elicited mixed responses; 46.7% (*n* = 14) were satisfied with the dietary limitations, with similar satisfaction levels observed between males and females.

The use of dental appliances significantly affected eating habits, with 46.7% (*n* = 14) of the children experiencing excessive salivation. This issue was more prevalent among male participants (66.7%, *n* = 10; *P* = 0.028). Additional details on discomfort associated with appliance therapy, including effects on eating habits and sleep disturbances, are outlined in Table 4.

**Table 4:** Functional aspect

Q. NO.	Questions	Answers	*N*	%
1	How satisfied are you with your speech?	Very satisfied	1	3.3
		Satisfied	8	26.7
		No feelings	4	13.3
		Dissatisfied	15	50.0
		Very dissatisfied	2	6.7
2	How has your speech been affected by the treatment provided by your pediatric dentist?	Improved	16	53.3
		Worsened	5	16.7
		Remains the same	9	30.0
3	Have you been to a speech therapist before? If yes, how satisfied are you with the results of the speech therapy you had?	Never visited a speech therapist	18	60
		Very satisfied	0	0
		Satisfied	6	20.0
		No feelings	4	13.3
		Dissatisfied	2	6.7
		Very dissatisfied	0	0
4	With the ongoing treatment, do you have any dietary restrictions? If yes, how satisfied are you with the same?	Very satisfied	0	0
		Satisfied	14	46.7
		No feelings	6	20.0
		Dissatisfied	8	26.7
		Very dissatisfied	2	6.7
5	How did the treatment provided by your pediatric dentist affect your eating habits?	Excessive Salivation	14	46.7
		Difficulty in chewing	9	30.0

The study highlighted several emotional and social challenges faced by children with CLP. Over half of the participants (56.7%, *n* = 17) reported difficulty with oral recitation at school due to compromised speech, which negatively impacted their confidence among peers. This issue was more pronounced among adolescents aged 12 years or older (75%, *n* = 9; *P* = 0.098) and was universally reported by participants attending government schools (100%, *n* = 6; *P* = 0.032).

A subset of children (16.7%, *n* = 5) missed school due to dental appointments, which affected their academic performance and concentration. Additionally, 46.7% (*n* = 14) felt less confident compared to their peers after wearing intraoral appliances, with higher incidences reported among adolescents (50%, *n* = 6) and male participants (53.3%, *n* = 8).

Teasing or bullying due to wearing dental appliances was reported by 26.7% (*n* = 8) of the children, predominantly among males under 12 years of age. Furthermore, 23.3% (*n* = 7), particularly females over 12 years old, expressed hesitation in engaging in physical activities due to fear of facial injuries that could exacerbate scarring, impacting their self-esteem and concerns about aesthetics. These emotional and social well-being findings are detailed in Table 5.

**Table 5:** Emotional aspect

Q. NO.	Questions	Answers	*N*	%
1	Were your school results affected during the course of treatment done by your pediatric dentist? If yes, what could be the probable reason?	Often missing school due to dental appointments	5	16.7
		Unable to concentrate into studies	5	16.7
		Difficulty in oral recitation due to compromised speech	17	56.7
		Feel tired throughout the day	1	3.3
2	Did you feel that during the course of treatment done by your pediatric dentist, it was more difficult for you to make friends?	Yes	3	10.0
		No	27	90
3	Do you feel that wearing an appliance made you less confident than your friends?	Yes	14	46.7
		No	16	53.3
4	Have you been teased because of your appliance given by your pediatric dentist?	Yes	8	26.7
		No	22	73.3
5	Has the appliance therapy prevented you from participating in any school sports activities?	Yes	7	23.3
		No	23	76.7

## Discussion

CLP are among the most prevalent congenital anomalies worldwide, leading to significant functional and aesthetic challenges that affect speech, hearing, dental health and psychological well-being [9]. Comprehensive treatment for CLP is intricate, involving multiple disciplines including surgery, orthodontics, speech therapy, and psychological support and often extends over several years. Understanding the perspectives and concerns of patients and their families is crucial for enhancing care and improving treatment outcomes [10]. This questionnaire-based study aimed to explore the treatment expectations and concerns of individuals with CLP, providing valuable insights to inform clinical practices better and meet the needs and expectations of patients and their caregivers.

Our study highlights the pivotal role of pediatric dentists within the multidisciplinary team managing CLP patients. 76.6% of participants were first introduced to a pediatric dentist by surgeons involved in their surgical care. This finding is in accordance with previous research indicating that surgeons are typically the initial point of contact for CLP patients, performing primary surgeries such as lip closure and palate repair [11]. The early involvement of pediatric dentists is critical, as they provide ongoing oral healthcare during three essential developmental stages: the newborn period, the primary and mixed dentition stages, and adolescence. Pediatric dentists contribute significantly to the comprehensive management of CLP by addressing dental anomalies, guiding craniofacial growth, and coordinating care with other specialists. Early engagement between families and pediatric dentists facilitates the establishment of trust and ensures that oral health considerations are integrated into the overall treatment plan from the outset [4, 12].

In our cohort, 90% of participants were undergoing fixed appliance therapy, with 53.3% receiving arch expansion using a quad helix and 26.7% undergoing skeletal expansion with a face mask. These interventions are standard in managing maxillary deficiencies and dental arch discrepancies commonly seen in CLP patients. The high prevalence of fixed appliance therapy highlights the necessity for patient compliance and the challenges associated with long-term treatment adherence [11, 13].

The significant proportion of participants (66.7%) aged over 12 years who had been in treatment for more than three years emphasizes the extended nature of CLP management. This prolonged treatment duration necessitates sustained motivation and support for patients and families, underscoring the importance of effective communication and patient education by healthcare providers.

Aesthetic considerations were prominent among participants, with 56.7% expressing dissatisfaction with their facial appearance. Female participants reported higher levels of concern (66.7%) compared to males, reflecting societal pressures and gender differences in self-perception. Despite ongoing treatments, 70% of patients remained primarily concerned about the appearance of their teeth, indicating that dental aesthetics are a critical factor influencing patient satisfaction. These findings are consistent with previous studies demonstrating that facial appearance significantly affects the psychological well-being and social integration of individuals with CLP [7, 10]. Raghavan et al. [7]. highlighted that dental aesthetics profoundly influence self-esteem and social confidence, especially during adolescence. Therefore, addressing aesthetic outcomes should be a priority in treatment planning, with clear communication regarding expected results and timelines to manage patient expectations effectively.

Speech difficulties were reported by 50% of participants, with a higher prevalence among children who were under 12 years and females. Despite the challenges, 53.5% believed that treatment by the pediatric dentist had improved their speech. However, a notable gap was identified, as 60% had not received formal speech therapy, and only 20% of those who did were satisfied with the outcomes. This shows a critical need for integrated speech therapy services within the multidisciplinary care framework. Early and effective speech intervention is vital, as approximately half of the children with CLP will require speech and language therapy at some point. Pediatric dentists can play a crucial role in facilitating referrals to speech therapists and reinforcing the importance of addressing speech issues as part of comprehensive care [13]. Lane et al. [14] emphasized that early naturalistic therapies positively impact speech development in children with CLP, highlighting the benefits of timely intervention.

Cleft care models in Scandinavian countries offer valuable insights into the benefits of centralized, structured health systems. In Sweden, cleft care is coordinated through six regional cleft teams, with structured early interventions embedded in national health services. These include standardized follow-up of speech, hearing, and facial growth, all systematically monitored via the Swedish CLP registry, ensuring consistent care quality and outcome evaluation across the country [15]. Norway implements a centrally coordinated cleft care system through two national centers that adhere to standardized treatment protocols. These centers provide multidisciplinary follow-up for all children with CLP from birth through adulthood, reflecting a commitment to long-term, integrated care [16]. These systems report high parental satisfaction and early speech outcomes, unlike the fragmented, referral-dependent approach observed in many Indian settings. Similarly, in Brazil and Thailand, community-based cleft care models anchored in public hospitals and supported by regional outreach have effectively reduced access gaps by integrating surgical, dental, and speech services within the local healthcare infrastructure [17, 18].

 The low uptake of speech therapy in our cohort stems not only from availability issues but also from socio-cultural barriers such as stigma, parental unawareness, and school absence concerns. This is in line with previous Indian studies that report poor awareness of cleft etiology among parents, prevailing superstitions, and reluctance to seek speech services due to limited understanding of its importance or fear of being judged. Markus et al. [19] reported that fewer than 30% of children attended speech therapy after surgical repair despite high parental satisfaction with surgical outcomes. The study highlighted widespread parental unawareness and misconceptions regarding cleft etiology, with only 7% of caregivers able to confidently explain the condition. Prevailing superstitions, such as beliefs linking clefts to maternal actions during eclipses, contribute to significant stigma, social isolation, and reluctance to seek speech services due to fear of judgment. Dvivedi and Dvivedi [20] documented that in the Sub-Himalayan Garhwal region, over 70% of parents were illiterate and most families belonged to low socioeconomic strata, limiting access to healthcare information and early intervention. The study also described the persistence of social stigma, with affected children often derogatorily labeled, and cultural misconceptions framing clefts as divine curses, which delay treatment and reduce engagement with speech therapy. Together, these studies underscore that socio-cultural factors including stigma, limited knowledge, superstitions, and economic hardship critically impede the utilization of speech therapy and comprehensive cleft care in India. Addressing these multifaceted barriers through targeted education and community support is essential to improving speech outcomes and overall quality of life for affected children. Kumar et al. [21] found that many parents were unaware of the need for continued speech therapy after surgery and lacked guidance from healthcare providers. Some had visited multiple hospitals without receiving a speech referral, and many were deterred by travel costs, lack of trained personnel, or concerns about interrupting schooling. Cultural stigma and fear of social judgment further contributed to therapy discontinuation. Together, these studies underscore that socio-cultural factors including stigma, limited knowledge, superstitions, and economic hardship critically impede the utilization of speech therapy and comprehensive cleft care in India. Addressing these multifaceted barriers through targeted education and community support is essential to improving speech outcomes and overall quality of life for affected children.

Dietary restrictions associated with appliance therapy were another concern, with 33.4% of participants expressing dissatisfaction. These restrictions are essential to prevent damage to appliances and maintain oral health. Continuous dietary counseling by pediatric dentists is necessary to ensure adherence and minimize the impact on the quality of life [4]. En et al. [22] noted that patients often require ongoing support to adapt to dietary changes during fixed appliance treatment.

Participants reported initial side effects of appliance therapy, including excessive salivation (46.7%), frequent ulceration, and halitosis. Excessive salivation is commonly due to mechanical stimulation from the appliance acting as a foreign object in the mouth. Studies have shown that the placement of fixed orthodontic appliances can increase saliva flow rates and alter salivary composition, likely due to heightened mechanosensory stimulation [23, 24].

Oral ulcerations often result from appliance irritation, necessitating adjustments during follow-up visits to alleviate discomfort. Halitosis can occur due to poor oral hygiene and increased plaque retention around appliances. Santonocito and Polizzi highlighted that appliance therapy can alter the oral microbiota, emphasizing the importance of rigorous oral hygiene practices and regular dental check-ups [25].

93.3% of patients did not experience alterations in sleep quality due to appliance therapy, aligning with previous findings. This suggests that while initial discomforts are common, they may not significantly impact sleep patterns in the long term.

The emotional and social well-being of children with CLP is profoundly affected by their condition. In our study, 56.7% of participants reported difficulty with oral recitation at school due to compromised speech, leading to reduced confidence among peers. Adolescents were particularly affected, with 75% expressing this issue. Additionally, 46.7% felt less confident after wearing intraoral appliances compared to their peers.

Bullying and teasing were reported by 26.7% of participants, primarily among males under 12 years old. These negative social experiences can lead to isolation, anxiety, and depression. Addressing these challenges requires a holistic approach that includes psychological support and interventions aimed at enhancing self-esteem and social skills. Turner et al. [26] advocated for counseling and social interaction skills training to improve the psychosocial outcomes for CLP patients. 23.3% of participants, particularly females over 12 years old, expressed hesitation in engaging in physical activities due to fear of facial injuries that could exacerbate scarring. This highlights the need for encouraging safe participation in sports and physical activities to promote social integration and physical health.

Parents of children with CLP often experience heightened psychological stress. The demands of managing a chronic condition, coordinating care among multiple specialists, and supporting their child’s emotional needs can be overwhelming. Incorporating family-centered care approaches and providing resources for parental support are essential components of comprehensive CLP management [27, 28].

Pediatric dentists can facilitate connections between families and clinical psychologists or support groups to alleviate stress and improve coping strategies. Enhancing public awareness and education about CLP can also reduce stigma and promote a more inclusive environment for affected children [4, 12].

This study has several limitations. The reliance on self-reported data introduces the potential for response bias, as participants may have provided socially desirable answers or may not accurately recall past experiences. The sample size, while adequate for an exploratory study, may not be large enough to generalize the findings to all CLP patients. The cross-sectional design does not allow for assessment of changes over time or the long-term effects of interventions on quality of life and psychological well-being. Future research should include larger, more diverse populations and employ longitudinal designs to track outcomes over time. Qualitative studies could also provide deeper insights into the experiences of patients and families, informing more tailored interventions.

## Conclusion

This study emphasizes the diverse concerns and treatment outlooks of children with cleft lip and palate, highlighting the pivotal role of pediatric dentists within a multidisciplinary care approach. Effective management of CLP requires a focus not only on addressing aesthetic and functional needs but also on supporting the emotional and social well-being of patients. Early intervention, enhanced collaboration among healthcare providers, and personalized, integrated care are essential to meet the unique and evolving needs of CLP patients and their families. By actively considering the perspectives of both patients and caregivers, clinicians can create customized treatment plans that enhance satisfaction and contribute to a higher quality of life for children navigating CLP related challenges.

## Supplementary Information

Supplementary Material 1.

## Data Availability

The data that support the findings of this study are available from the corresponding author upon reasonable request.

## Abbreviation

CLP: Cleft lip and palate
