# Supplementary material for: Treatment perspectives and concerns among pediatric cleft lip and palate patients: a cross-sectional study
Source: BMC Oral Health. 2025 Aug 12;25:1323. doi: 10.1186/s12903-025-06693-w (PMC12345027; doi:10.1186/s12903-025-06693-w)
Supplement: Supplementary file 1 — Supplementary Material 1. [file 12903_2025_6693_MOESM1_ESM.docx]

**Questionnaire**

**Part 1: Sociodemographic Details**

**1. Age:** ________ years

**2. Gender**
☐ Male
☐ Female

**3. Type of School Attended**
☐ Government
☐ Private

**4. Socio-Economic Status**
☐ Upper (I)
☐ Upper Middle (II)
☐ Lower Middle (III)
☐ Upper Lower (IV)
☐ Lower (V)

**Part 2: Treatment Aspect**

**1. Who referred you to a pediatric dentist for cleft lip and palate treatment?**
☐ Surgeon
☐ Orthodontist
☐ Speech Therapist
☐ Others: ____________

**2. How long have you been visiting a pediatric dentist?**
☐ Less than 1 year
☐ More than 1 year
☐ More than 2 years
☐ More than 3 years

**3. What treatment are you undergoing at present?**
☐ Arch expansion
☐ Skeletal correction
☐ Teeth alignment
☐ Prosthetic rehabilitation

**4. What type of appliance are you using now?**
☐ Fixed appliance
☐ Removable appliance

**5. Which group has been most important in your care?**
☐ Surgeon
☐ Orthodontist
☐ Pediatric Dentist
☐ Speech Therapist

**Part 3: Aesthetic Aspect**

**1. How satisfied are you with your facial appearance at the moment?**
☐ Very satisfied
☐ Satisfied
☐ No feelings
☐ Dissatisfied
☐ Very dissatisfied

**2. Is there a noticeable change in your appearance from the beginning of your treatment until now?**
☐ Yes  ☐ No
**If yes, which part of your face has changed?**
☐ Nose
☐ Lips
☐ Jaws
☐ Teeth

**3. Which part of your face is your main concern at present?**
☐ Nose
☐ Lips
☐ Jaws
☐ Teeth

**Part 4: Functional Aspect**

**1. How satisfied are you with your speech?**
☐ Very satisfied
☐ Satisfied
☐ No feelings
☐ Dissatisfied
☐ Very dissatisfied

**2. How has your speech been affected by the treatment provided by your pediatric dentist?**
☐ Improved
☐ Worsened
☐ Remains the same

**3. Have you been to a speech therapist before? If yes, how satisfied are you with the results of the speech therapy you had?**
☐ Never visited a speech therapist
☐ Very satisfied
☐ Satisfied
☐ No feelings
☐ Dissatisfied
☐ Very dissatisfied

**4. With the ongoing treatment, do you have any dietary restrictions? If yes, how satisfied are you with the same?**
☐ Very satisfied
☐ Satisfied
☐ No feelings
☐ Dissatisfied
☐ Very dissatisfied

**5. How did the treatment provided by your pediatric dentist affect your eating habits?**
☐ Excessive salivation
☐ Difficulty in chewing

**Part 5: Emotional Aspect**

**1. Were your school results affected during the course of treatment done by your pediatric dentist? If yes, what could be the probable reason?**
☐ Often missing school due to dental appointments
☐ Unable to concentrate into studies
☐ Difficulty in oral recitation due to compromised speech
☐ Feel tired throughout the day

**2. Did you feel that during the course of treatment done by your pediatric dentist, it was more difficult for you to make friends?**
☐ Yes
☐ No

**3. Do you feel that wearing an appliance made you less confident than your friends?**
☐ Yes
☐ No

**4. Have you been teased because of your appliance given by your pediatric dentist?**
☐ Yes
☐ No

**5. Has the appliance therapy prevented you from participating in any school sports activities?**
☐ Yes
☐ No
